# Racism and racial health inequity: four theories for public health

**DOI:** 10.1093/pubmed/fdag005

**Published:** 2026-01-30

**Authors:** Caroline Parker, Adeola Agbebiyi, Aine Fuller, Maddy Gupta-Wright, Sandra Husbands, John Licorish, Safia Marcano, Lee Pinkerton, Anne Pordes Bowers, Melissa Parker

**Affiliations:** Department of Anthropology, University College London, 14 Taviton Street, London, WC1H 0BW, UK; London Borough of Newham, Newham Dockside, 1000 Dockside Road, London E16 2QU, UK; London Borough of Newham, Newham Dockside, 1000 Dockside Road, London E16 2QU, UK; London Borough of Ealing, Ealing Council, Perceval House, 14-16 Uxbridge Road, Ealing W5 2HL, UK; London Borough of Hackney, Hackney Service Centre, 1 Hillman Street, London, E81 DY, UK; London Borough of Brent, Brent Civic Centre, Engineers Way, Wembley, HA9 0FJ, UK; London Borough of Hackney, Hackney Service Centre, 1 Hillman Street, London, E81 DY, UK; London Borough of Hackney, Hackney Service Centre, 1 Hillman Street, London, E81 DY, UK; London Borough of Newham, Newham Dockside, 1000 Dockside Road, London E16 2QU, UK; Department of Global Health and Development, London School of Hygiene & Tropical Medicine, 15-17 Tavistock Place, London WC1H 9SH, UK

Virtually all public health practitioners acknowledge the need to combat racism. Yet very few public health initiatives in the United Kingdom (UK) are rooted in social scientific ideas offering testable, adaptable frameworks for understanding and dismantling racial inequality. This reluctance does not stem from a lack of awareness of the causes of racial disparities—such as European colonialism, slavery, and their ongoing reverberations—but from the field’s focus on immediate, practical responses to urgent public health issues. As a result, social scientific theories—especially those exploring how ‘race’ is constructed and racial inequality perpetuated—are unevenly integrated into practice. But the social sciences offer precisely the concepts that are needed for understanding how racial categories are constructed and how they come to normalize racial health inequities, including those generated by public health.

This article identifies four key social theories essential to combatting racism and racial health inequity. These theories emerged when the London Association of Directors of Public Health (ADPHL) engaged two anthropologists, Dr Parker and Professor Parker, to help develop their anti-racism strategy. Over the past year, we collaborated through discussions and critically examined literature in critical race theory, Black studies, race and ethnicity studies, and the anthropology of white supremacy. Together, these sources form a body of conceptual tools to help public health dismantle racism and disparities.

The first theory is *racial formation*, introduced by Omi and Winant (1986) in *Racial Formation in the United States*. Now central to race and ethnicity studies, it offers a framework for understanding how racial categories are historically produced and maintained.

A key insight is that ‘race’ (along with ethnicity, color, and caste) is an historically produced power relationship, not an inherent quality, and these power relationships are constantly changing. Take the distinction between ‘race’ and ‘ethnicity.’ Whereas textbooks often define ‘race’ as categorizing people by physical traits—such as skin colour, facial features, and hair texture—‘ethnicity’ tends to be defined through reference to shared culture, history, language, and religion. However, what counts as ‘race’ varies internationally.^1^ ‘White’ is classified as a race in the US census but as an ethnicity in the UK census. This international variation in how ‘race’ and ‘ethnicity’ are defined reflects the fact that human variation is not inherently organized into universal, timeless fixed groups. In fact, someone considered ‘mixed race’ in the United Kingdom may be ‘Black’ in the United States, ‘White’ in Brazil, or, if we rewind just a few decades, ‘coloured’ in the UK census of the 1970s.^2^ Racial and ethnic categories are not scientific descriptions. They are power relationships imposed on human diversity in different ways at different historical moments.

While most public health practitioners recognize race as a social construct, few can articulate how their racial and ethnic categories came into being. Why, for instance, has the United Kingdom long used the label ‘BAME,’ but not ‘WME’ (White Majority Ethnic)? Why does the current UK census distinguish four Asian ethnicities (Indian, Pakistani, Bangladeshi, Chinese) while grouping people from the remaining forty-four Asian countries as ‘any other Asian background’? Similarly, people from fifty-four African nations are grouped as a single ‘Black African’ ethnicity. Social scientists have thoroughly explored the histories of racial and ethnic categories in Britain and across its empire, and we encourage public health practitioners to read their work.^3–5^ The key point is that without understanding the history behind classifications, contemporary racial categories can seem ‘natural’, leading efforts to address health inequities from a flawed premise: that some timeless phenomena called ‘race’ or ‘ethnicity’ directly impacts health. This assumption fosters inaccurate theories attributing inequities to biological traits or overly simplistic cultural differences.

The second concept is *global white supremacy*—an international power system rooted in the ideology that white people (broadly defined) are biologically or culturally superior to everyone else. This ideology normalizes shorter life expectancies and lower social status for other racial groups, often through pseudo-biological and cultural justifications. Anthropologists Aisha Beliso–De Jesus and Jemima Pierre^6^ argue that white supremacy has long been mischaracterized as the domain of neo-Nazis and extremists. In reality, it is a far more insidious ideology that permeates society and shapes institutions across medicine, public health, government, and democracy.

While public health frequently calls to combat racism, its acknowledgement of white supremacy has been strikingly timid. Despite hundreds of studies on racial health disparities, very few public health researchers name it. But social scientists recognize white supremacy at play when researchers ‘control for’ variables like lower income, education, and neighborhood resources of ethnoracial minorities. By ‘controlling for class,’ researchers accept racial inequality as ‘natural’ rather than as a power relationship that itself requires explanation. This naturalization of racial inequality narrows public health’s focus to behavioral, biological, or cultural factors. For example, Black Britains’ higher COVID-19 mortality is attributed to pre-existing conditions, while Pakistani mortality is ascribed to overcrowded housing. Though both factors may mediate disparities, they are not underlying causes. Accepting race-class hierarchies as normal—such as the 63% income gap favoring white households in the United Kingdom^7^—is itself white supremacist thinking. Denaturalizing racial-class hierarchies by naming white supremacy is therefore essential to understanding and dismantling racial health inequities.

A third theory is the *invisibility of whiteness*. Like ‘race,’ whiteness is a social construct, but it is often perceived as an absence of race. Daniels and Schultz^8^ observe, ‘a defining feature of whiteness… is the absence or unmarked invisibility of “white” as a racial category.’ This is evident in how ethnic disparities are represented. For decades, the health of different ethnic groups in Britain has been portrayed with the ‘white British’ population as the normal baseline. During the COVID-19 pandemic, Public Health England’s graphs repeatedly depicted the White British male population as the standard, the reference point against which mortality rates of other ethnic groups were compared (see Fig. 1).

**Figure 1:**
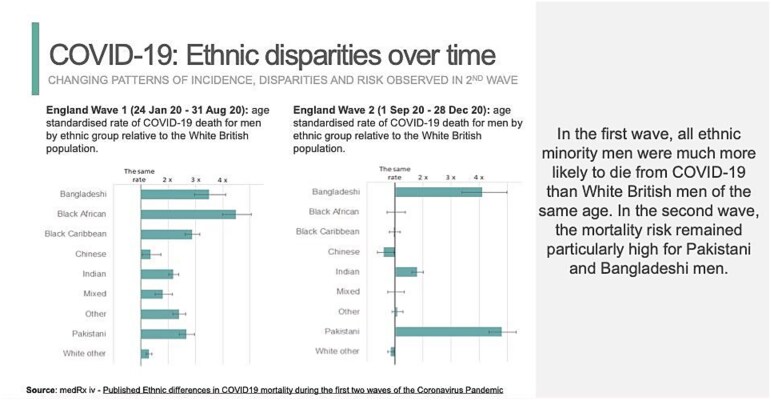
Fenton, Kevin. 2021. ‘COVID-19, Health Inequalities and Recovery’. In Public Health England, protecting and improving the Nation’s Health.

Presenting ethnic disparities with white British people as the baseline involves significant omissions. By positioning ‘British whites’ as the default, it erases that ‘white British’ is itself an ethnic category. Treating ‘white British’ as an unmarked raceless category implies only other groups need an ‘ethnic’ explanation for their health, shielding white British people from recognizing how racism in white supremacist societies impacts their health, often in protecting ways.

How public health practitioners share data on racial health disparities is crucial to combating racism. Guidelines increasingly recommend moving away from standardizing around whiteness.^9^^,^^10^ They advise comparing groups to the overall population rather than defaulting to ‘whites,’ disaggregating ‘white’ into constituent categories, and acknowledging that racial and ethnic categories are socially constructed. Guidelines also recommend explicitly theorizing how ‘race’ influences health through embodied racism and avoiding framing it as a standalone risk factor.^11^ Without this clarity, public health risks essentializing racial categories, normalizing disparities, and perpetuating the invisibility of whiteness—an aspect of white supremacy—by attributing minorities’ premature deaths to ‘race’ or ‘ethnicity’ and privileging flawed biological or cultural explanations.

Our fourth concept is *racial gaslighting*. In the 1944 film Gaslight, a husband manipulates his wife into doubting her sanity by altering her environment and dismissing her perceptions. Davis and Ernst^12^ use ‘racial gaslighting’ to describe analogous efforts to undermine the perceptions of racial minorities, making them question their own experiences. This is evident in public health debates about race and ‘medical mistrust.’ In recent years, ‘mistrust’ has become a central focus of UK public health policy. Hundreds of papers analyse racial differences in ‘mistrust’ in medicine, science, and healthcare services, including attitudes toward COVID-19 vaccines.^13^^,^^14^ Many link mistrust and vaccine hesitancy to ethnicity, yet these studies often say little about what underlies or justifies such perceptions and experiences.

Sociologist Ruja Benjamin^15^ notes that such framing has allowed ‘Black distrust’ to be widely accepted as a cultural trait unique to Black communities. It has also produced racial codes, like “low-trust communities,”^16^ where absence of trust defines racialized health profiles. Attributing racial disparities in ‘medical mistrust’ to shorter life expectancies among racial minorities amounts to racial gaslighting. By emphasizing attitudes—without examining why Black women in the United Kingdom are nearly four times more likely to die in childbirth, or why Black babies are nearly twice as likely to be flagged for NHS safety investigations^17^—racial gaslighting dismisses the genuine concerns of racialized communities and obscures the reality of a white supremacist power structure.

These four theories do not encompass all social theories of race relevant to public health. Many other frameworks help explain how racial health inequity is produced, visualized, interpreted, and often naturalized, including ‘technologies of race,’ ‘technologies of whiteness,’ ‘racial common sense,’ and the ‘antipolitics of trust talks’.^18–20^ Nevertheless, these four theories enable public health practitioners to critically reflect on and resist the naturalization of racial inequality. Social sciences must equip practitioners with frameworks that acknowledge white supremacy and combat racism. Interdisciplinary collaboration is essential to develop a comprehensive understanding of race, racism, and racial health inequities, promoting effective, equitable public health strategies.

## Key Messages

- Critical engagement with social sciences—including critical race theory, Black studies, race and ethnicity studies, and the anthropology of white supremacy—is essential for developing effective, anti-racist public health strategies that promote racial health equity and justice.
- Most public health initiatives to combat racism are not sufficiently grounded in social scientific theories of racism.
- Four key theories—racial formation, white supremacy, the invisibility of whiteness, and racial gaslighting—can guide efforts to dismantle racism and address racial health inequities.
- Tackling racial health inequities requires that we stop taking race-class hierarchies as the ‘natural' order of things and fundamentally rethink how we measure and represent racial inequality.

## Notes

**Caroline Parker**, Lecturer of Anthropology

**Adeola Agbebiyi**, Assistant Director of Public Health

**Aine Fuller**, Assistant Public Health Strategist

**Maddy Gupta-Wright**, Co-director, Consultant in Public Health

**Sandra Husbands**, Director of Public Health and ADPHL Programme Co-chair for Tackling Racism and Inequality

**John Licorish**, Consultant in Public Health

**Safia Marcano**, ADPHL Programme Manager

**Lee Pinkerton**, ADPHL Communication and Engagement Officer

**Anne Pordes Bowers**, Strategic Lead for Community Public Health and Inclusive Economy

**Melissa Parker**, Professor of Medical Anthropology
